# Preterm Birth Associated With Group B *Streptococcus* Maternal Colonization Worldwide: Systematic Review and Meta-analyses

**DOI:** 10.1093/cid/cix661

**Published:** 2017-11-06

**Authors:** Fiorella Bianchi-Jassir, Anna C Seale, Maya Kohli-Lynch, Joy E Lawn, Carol J Baker, Linda Bartlett, Clare Cutland, Michael G Gravett, Paul T Heath, Margaret Ip, Kirsty Le Doare, Shabir A Madhi, Samir K Saha, Stephanie Schrag, Ajoke Sobanjo-ter Meulen, Johan Vekemans, Craig E Rubens

**Affiliations:** 1 Maternal, Adolescent, Reproductive and Child Health Centre, London School of Hygiene & Tropical Medicine, United Kingdom;; 2 College of Health and Medical Sciences, Haramaya University, Dire Dawa, Ethiopia;; 3 Centre for Child and Adolescent Health, School of Social and Community Medicine, University of Bristol, United Kingdom;; 4 Departments of Pediatrics and Molecular Virology and Microbiology, Baylor College of Medicine, Houston, Texas;; 5 Department of International Health, Johns Hopkins Bloomberg School of Public Health, Baltimore, Maryland;; 6 Medical Research Council: Respiratory and Meningeal Pathogens Research Unit, and Department of Science and Technology/National Research Foundation: Vaccine Preventable Diseases, Faculty of Health Sciences, University of the Witwatersrand,Johannesburg, South Africa;; 7 Global Alliance to Prevent Prematurity and Stillbirth, Seattle, Washington;; 8 Department of Obstetrics and Gynecology, University of Washington, Seattle;; 9 Vaccine Institute, Institute for Infection and Immunity, St George’s Hospital, University of London and St George’s University Hospitals NHS Foundation Trust, United Kingdom;; 10 Department of Microbiology, Faculty of Medicine, Chinese University of Hong Kong;; 11 Centre for International Child Health, Imperial College London, United Kingdom;; 12 National Institute for Communicable Diseases, National Health Laboratory Service, Johannesburg, South Africa;; 13 Bangladesh Institute of Child Health, Dhaka;; 14 National Center for Immunization and Respiratory Diseases, Centers for Disease Control and Prevention, Atlanta, Georgia;; 15 Bill & Melinda Gates Foundation, Seattle, Washington;; 16 World Health Organization, Geneva, Switzerland; and; 17 Department of Global Health, University of Washington, Seattle

**Keywords:** group B *Streptococcus*, preterm delivery, preterm labor, colonization, bacteriuria

## Abstract

**Background:**

Preterm birth complications are the leading cause of deaths among children <5 years of age. Studies have suggested that group B *Streptococcus* (GBS) maternal rectovaginal colonization during pregnancy may be a risk factor for preterm delivery. This article is the fifth of 11 in a series. We aimed to assess the association between GBS maternal colonization and preterm birth in order to inform estimates of the burden of GBS.

**Methods:**

We conducted systematic literature reviews (PubMed/Medline, Embase, Latin American and Caribbean Health Sciences Literature [LILACS], World Health Organization Library Information System [WHOLIS], and Scopus) and sought unpublished data from investigator groups on the association of preterm birth (<37 weeks’ gestation) and maternal GBS colonization (GBS isolation from vaginal, cervical, and/or rectal swabs; with separate subanalysis on GBS bacteriuria). We did meta-analyses to derive pooled estimates of the risk and odds ratios (according to study design), with sensitivity analyses to investigate potential biases.

**Results:**

We identified 45 studies for inclusion. We estimated the risk ratio (RR) for preterm birth with maternal GBS colonization to be 1.21 (95% confidence interval [CI], .99–1.48; *P* = .061) in cohort and cross-sectional studies, and the odds ratio to be 1.85 (95% CI, 1.24–2.77; *P* = .003) in case-control studies. Preterm birth was associated with GBS bacteriuria in cohort studies (RR, 1.98 [95% CI, 1.45–2.69]; *P* < .001).

**Conclusions:**

From this review, there is evidence to suggest that preterm birth is associated with maternal GBS colonization, especially where there is evidence of ascending infection (bacteriuria). Several biases reduce the chance of detecting an effect. Equally, however, results, including evidence for the association, may be due to confounding, which is rarely addressed in studies. Assessment of any effect on preterm delivery should be included in future maternal GBS vaccine trials.

## Teaser

Complications of preterm birth are the leading cause of deaths in children aged <5 years. Group B *Streptococcus* may be a preventable and important contributor to this burden.

There are approximately 15 million preterm (<37 weeks’ gestation) births worldwide in a year; an estimated 11% of all live births [1]. Complications of preterm birth are the most common direct cause of death in children <5 years of age, accounting for 15% of all child deaths and 35% of all neonatal deaths worldwide [2–4]. Preterm birth is also an indirect contributor in approximately half of all neonatal deaths, through interaction with other direct causes such as neonatal infection [1]. Beyond this, preterm birth can result in long-term disability among survivors, including neurodevelopmental and cognitive disorders, visual and hearing impairment, motor disorders, risk of severe infections, and long-term metabolic, cardiovascular, and mental health disorders [5].

Preterm birth is a risk factor for invasive bacterial disease, including group B *Streptococcus* (GBS; *Streptococcus agalactiae*) infections in the newborn [6–8]. However, evidence for the association between maternal colonization or infection and preterm birth, with bacteria such as GBS, is unclear [9, 10]. A previous systematic review investigating the association between maternal GBS colonization and preterm birth demonstrated conflicting findings [11]. Associations were detected between maternal GBS colonization and preterm birth in cross-sectional and case-control studies, when cultures were performed at delivery, but not in longitudinal cohort studies, when cultures were performed earlier in pregnancy.

The putative mechanism for preterm birth from colonization and/or infection relates to specific changes in bacterial flora in the vagina and, in some cases, overgrowth that may increase the risk of ascending infection through the cervix, resulting in bacterial infection of fetal membranes and decidua causing: (1) secretion of proteases that degrade the extracellular matrix within the fetal membranes, and/or (2) a host inflammatory response with cytokine production, and stimulation of prostaglandin and protease synthesis, which increases uterine contractility and results in preterm delivery [9, 12, 13]. This is supported by recent work in animal models which shows that GBS produces extracellular membrane vesicles that, through certain virulence factors and toxins, lead to extraplacental membrane weakening, degradation of collagen, and preterm birth [14]. The authors also demonstrated that the association with preterm birth was independent of having culture of GBS present in the amniotic fluid; that is, the extracellular membrane vesicles led to a “sterile intra-amniotic inflammation” that induced preterm birth [14].

**Figure 1.**
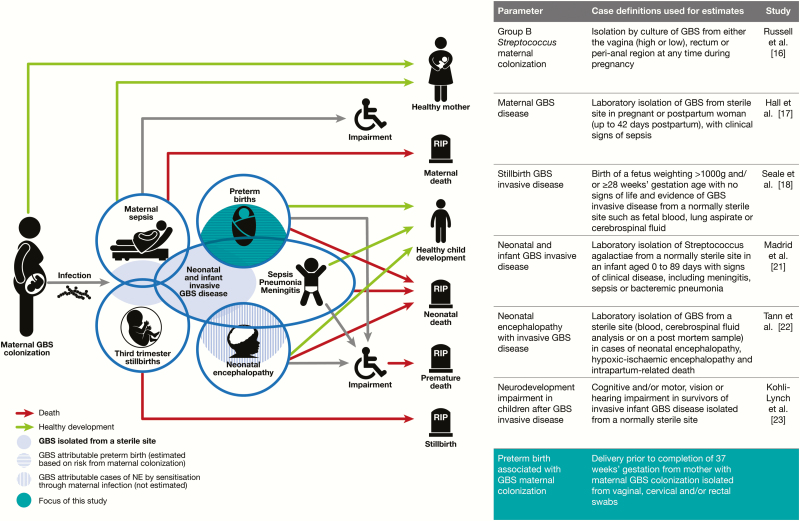
Preterm birth in the disease schema for group B streptococcal disease, as described by Lawn et al [15]. Abbreviations: GBS, group B *Streptococcus*; NE, neonatal encephalopathy.

This article assesses the risk of preterm birth associated with maternal GBS colonization (Figure 1) and is part of a supplement estimating the burden of GBS disease in pregnant women, stillbirths, and infants, which is important in terms of public health policy, particularly vaccine development [15]. The supplement includes systematic reviews and meta-analyses on GBS colonization, and adverse outcomes associated with GBS around birth [16–23], which form input parameters used for estimates of the burden of GBS, partly through a compartmental model [24].

The specific objectives of this article are as follows:

1. To undertake a comprehensive and systematic literature review and meta-analyses to assess (*i*) the association between maternal GBS colonization and preterm birth, and (*ii*) the association between GBS isolated from urine or chorioamnion cultures and preterm birth;
2. To assess these data to inform estimates (if appropriate) for the contribution of preterm birth to the total burden of GBS in pregnancy for women, stillbirths, and infants;
3. To evaluate the gaps in the data and recommend how to improve the data on the association between maternal GBS colonization and preterm birth.

## METHODS

This article is part of a wider study protocol entitled “Systematic estimates of the burden of GBS worldwide in pregnant women, stillbirths and infants.” It was submitted for ethical approval to the London School of Hygiene & Tropical Medicine (reference citation 11966) and approved on 30 November 2016.

### Definitions

Preterm delivery is defined as delivery prior to completion of 37 weeks’ of gestation (measured by ultrasound, fundal height, or date of last menstrual period [LMP]). Preterm labor is defined as the occurrence of frequent uterine contractions (a specific number in a specific time period, eg, 1 every 5–8 minutes) plus cervical dilatation >1 cm and cervical effacement (≥50%) before 37 weeks’ gestation (details of study characteristics are shown in Supplementary Materials).

Maternal GBS colonization was defined as GBS isolated from vaginal, cervical, and/or rectal swabs from pregnant women. Studies reporting maternal GBS isolation from urine or chorioamnion cultures were analyzed separately.

### Search Strategy and Selection Criteria

We identified data through systematic review of the published literature and from an investigator group of clinicians, researchers, and relevant professional institutions worldwide. For this paper, we did systematic literature searches in Medline, Embase, Scopus, the World Health Organization Library Information System (WHOLIS), and Literature in the Health Sciences in Latin America and the Caribbean (LILACS) from 20 to 25 October 2016, and updated these on 6 February 2017. The search terms for preterm delivery were consistent with those used for estimating the burden of preterm birth [25], including variants of the terms “preterm birth,” “premature,” and “preterm labor,” and were combined with search terms for “GBS” or “*Streptococcus agalactiae*.” Medical subject heading (MeSH) terms were used where possible. The full list of search terms is presented in Supplementary Table 1. The search was limited to humans and there were no language or date restrictions. Case reports, case series, and reviews were excluded, as well as studies without an appropriate comparison group (studies that measured preterm delivery within a group of women in preterm labor) (Supplementary Table 2). We used snowball searches of article reference lists including reviews to identify additional studies.

One investigator performed the database search, screened for duplicates and screened titles for eligibility, and selected abstracts to assess their eligibility for inclusion. Two independent investigators (F. B. J. and M. K. L.) assessed the full-length articles previously selected to determine their inclusion and extracted data. Where there was discrepancy between 2 reviewers, a third investigator (A. S.) made the final decision.

Studies were assessed for bias using specific criteria (study site, sampling and laboratory methods for GBS detection, and measurement of gestational age), and the effects of these criteria were investigated in sensitivity analyses.

### Meta-analyses and Sensitivity Analyses

Data from each study were extracted into standard Excel forms and imported to Stata 14 software (StataCorp) for meta-analyses. We used random-effects meta-analyses to estimate risk ratios and odds ratios using the DerSimonian and Laird method [26]. Meta-analyses were done for each study design (case-control, cross-sectional, and cohort). For cohort and cross-sectional studies, a pooled risk ratio was calculated, and for case-control studies a pooled odds ratio was calculated.

We did sensitivity analyses to see if there was misclassification in the exposure or outcome resulting in bias. These were:

1. Exposure classification: reducing misclassification through increasing sensitivity of detection through sample site and laboratory method (rectovaginal sampling and broth enrichment) [16];
2. Exposure classification: to evaluate effect of using nonselective laboratory methods and cervical and upper vaginal sampling, which could reflect detection of more heavily colonized women;
3. Exposure classification: reducing misclassification through including only studies where mothers were reported to have not used antibiotics during pregnancy or at least 1 week before the culture sample was taken;
4. Exposure classification: timing of sample-taking, comparing samples which were taken in antenatal visits or at delivery;
5. Outcome classification: reducing misclassification by including only studies that described how gestational age (GA) was measured and if methods used were LMP, fundal height, and/or ultrasound;
6. Outcome classification: reducing overestimation of effect if relationship is nonlinear by excluding different thresholds for the definition of preterm (or if definition not specified).

## RESULTS

### Study Selection

**Figure 2.**
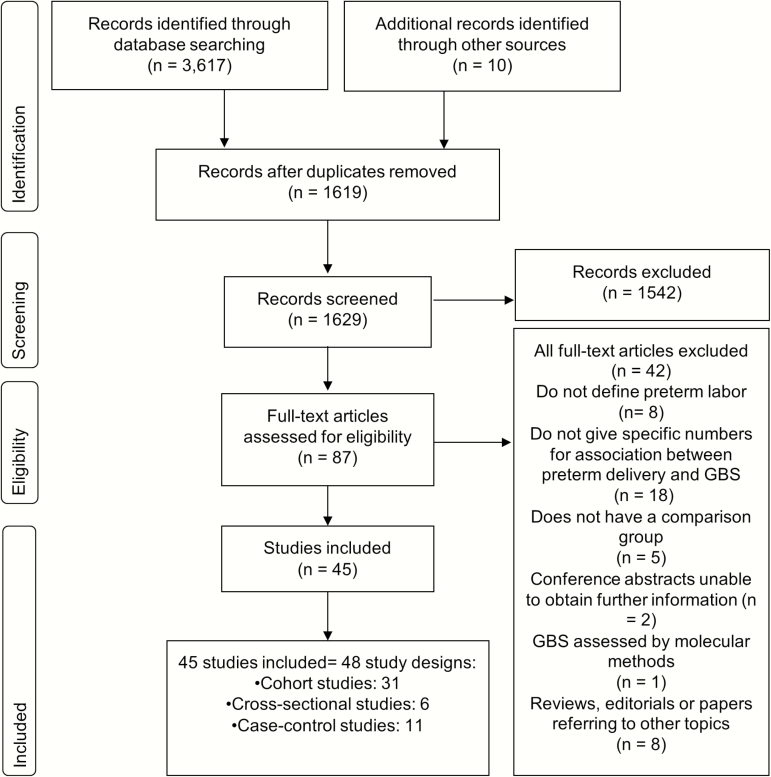
Search strategy and study selection for analyzing the potential association between maternal group B *Streptococcus* (GBS) colonization and preterm birth.

We identified 3617 records from databases of published literature; 1 unpublished dataset and 9 records were identified through snowball searches. After the selection process, 45 studies were included in this systematic review (Figure 2) (LeDoare, unpublished data) [27–70].

### Study Characteristics

**Figure 3.**
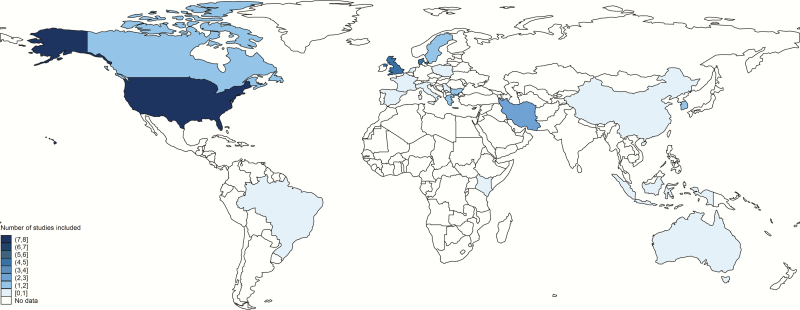
Countries where published and unpublished data were found for the analyses.

Most studies (33/45) were from developed countries, including 8 from the United States, and 22 from Europe. There were fewer studies from low- and middle-income contexts (12/45), including Africa (2), Middle-Eastern Asia (5), South-Eastern Asia (1), East Asia (3), and Latin America (1) (Figure 3) (LeDoare, unpublished data) [27, 31, 35, 41, 46, 48, 61–64, 68]. Of all studies, there were 11 case-control studies, 31 cohort studies (8 of which were retrospective cohorts), and 6 cross-sectional studies. Sometimes studies included >1 study design and we included the results for each in the appropriate meta-analysis [36, 46]. From these, 2 case-control studies and 9 cohort studies were included in the separate subanalysis on GBS bacteriuria.

Borders of countries/territories in map do not imply any political statement.

In terms of the exposure, the prevalence of maternal GBS colonization reported in studies ranged from 1.4% to 48.4% (median, 12.5%), and the prevalence of preterm birth ranged from 1.8% to 46.7% (median, 9.1%). Further details of the included studies are shown in Supplementary Table 3.

### Meta-analyses and Sensitivity Analyses

**Figure 4.**
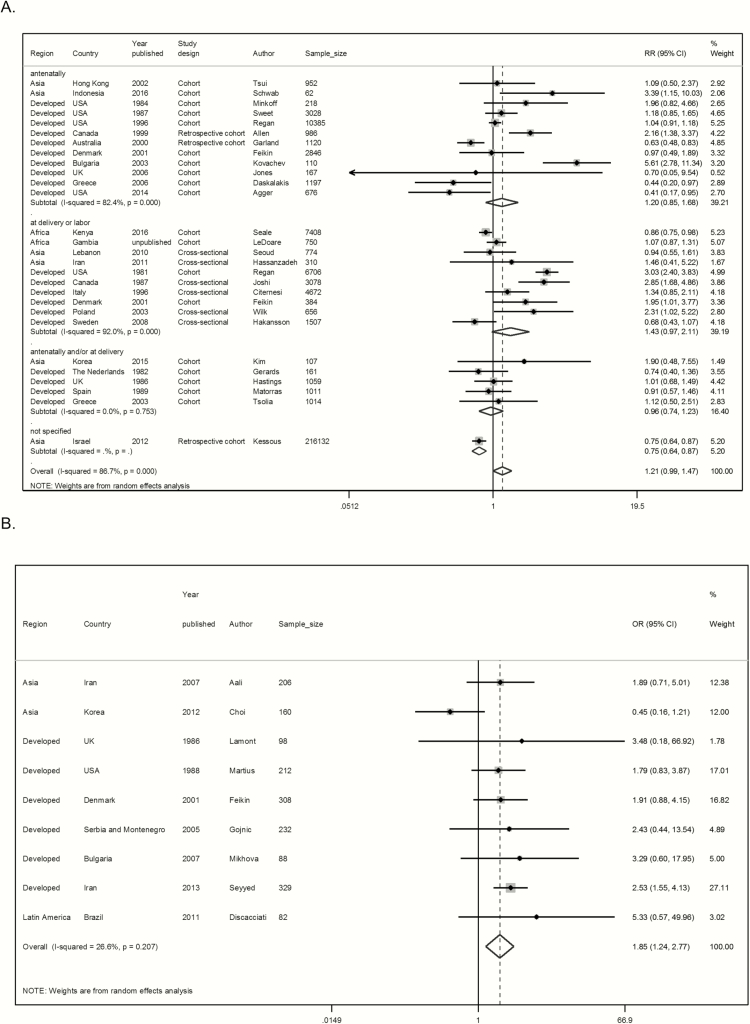
Pooled estimates of association between preterm birth and maternal group B *Streptococcus* (GBS) colonization, split by study design. *A*, Cohort or cross-sectional studies by time of maternal GBS screening. *B*, Case-control studies. Abbreviations: CI, confidence interval; OR, odds ratio.

There was some evidence of an association between maternal GBS colonization and preterm birth in cohort and cross-sectional studies (risk ratio [RR], 1.21 [95% confidence interval {CI}, .99–1.48]; *P* = .061) and in case-control studies (odds ratio [OR], 1.85 [95% CI, 1.24–2.77]; *P* = .003) (Figure 4). For the studies that used urine samples or other sources to identify patients as GBS carriers, there was strong evidence that maternal GBS bacteriuria was associated with preterm birth (RR, 1.98 [95% CI, 1.45–2.69], *P* < .001, n = 9; and OR, 1.97 [95% CI, .65–5.98], *P* = .232, n = 2, for cohort studies compared with case-control studies, respectively) (Supplementary Figures 1 and 2).

The results for all sensitivity analyses are detailed in Table 1 and summarized below.

**Table 1.** Pooled Estimates of Association Between Preterm Birth and Maternal Group B *Streptococcus* Colonization, According to Study Design, and Results From Sensitivity Analyses to Show Various Potential Biases in Exposure and Outcome

Meta-analysis/Sensitivity Analysis	Rationale		No. of Studies Included	Point Estimate	(95% CI)	*P* Value^a^
Cohort and cross-sectional studies	Main analysis from GBS isolated from vaginal, cervical, and/or rectal swabs from pregnant women		28	RR = 1.21	(.99–1.48)	.061
Exposure classification- (sampling and laboratory factors)	Reducing misclassification through increasing sensitivity of detection through sample site and laboratory method (rectovaginal sampling and broth enrichment)		12	RR = 1.02	(.85–1.22)	.862
Exposure classification (heavily colonized women)	Using nonselective laboratory methods and cervical and upper vaginal sampling to evaluate effect of detecting more heavily colonized women		8	RR = 1.65	(1.03–2.64)	.036
Exposure classification (prior antibiotic use)	Reducing misclassification through including only studies where mothers were reported to have not used antibiotics during pregnancy or at least 1 week before the culture sample was taken		4	RR = 1.48	(.61–3.62)	.387
Exposure classification (sample timing)	Timing of sample-taking, comparing samples that were taken in antenatal visits or at delivery	Antenatal screening	12	RR = 1.20	(.85–1.68)	.297
		At delivery or labor	10	RR = 1.43	(.97–2.11)	.071
		Antenatal and/or at delivery	5	RR = 0.96	(.74–1.23)	.731
Outcome classification (gestational age measurement)	Reducing misclassification by including only studies that described how gestational age was measured and if methods used were last menstrual period, fundal height, and/or ultrasound		9	RR = 1.14	(.79–1.65)	.493
Outcome classification (preterm definition)	Reducing overestimation of effect if relationship is nonlinear by excluding different thresholds for the definition of preterm (or if definition not specified)		23	RR = 1.07	(.92–1.25)	.388
Case-control studies	Main analysis from GBS isolated from vaginal, cervical, and/or rectal swabs from pregnant women		9	OR = 1.85	(1.24–2.77)	.003
Exposure classification (sampling and laboratory factors)	Reducing misclassification through increasing sensitivity of detection through sample site and laboratory method (rectovaginal sampling and broth enrichment)		3	OR = 1.35	(.33–5.60)	.676
Exposure classification (heavily colonized women)	Using nonselective laboratory methods and cervical and upper vaginal sampling to evaluate effect of detecting more heavily colonized women		4	OR = 2.08	(1.19–3.62)	.010
Exposure classification (prior antibiotic use)	Reducing misclassification through including only studies where mothers were reported to have not used antibiotics during pregnancy or at least 1 week before the culture sample was taken		5	OR = 2.32	(1.61–3.34)	<.001
Outcome classification (gestational age measurement)	Reducing misclassification by including only studies that described how gestational age was measured and if methods used were last menstrual period, fundal height, and/or ultrasound		3	OR = 1.86	(1.15–2.99)	.011
Outcome classification (preterm definition)	Reducing overestimation of effect if relationship is nonlinear by excluding different thresholds for the definition of preterm (or if definition not specified)		6	OR = 2.21	(1.59–3.08)	<.001

Abbreviations: CI, confidence interval; GBS, group B *Streptococcus*; OR, odds ratio; RR, risk ratio.

^a^ *P* value of significance test of RR = 1 or OR = 1.

1. Exposure classification (sampling and laboratory factors): lower point estimate for cohort and cross-sectional studies using rectovaginal sampling and nonselective media excluded. For case-control studies, no evidence of an association was found, in contrast to the initial analysis. However, as only 3 studies were included in this analysis, this result may be due to reduced power to detect the association (Table 1; Supplementary Figures 3 and 4).
2. Exposure classification (women with heavy colonization): Cohort studies using nonselective medium and sampling from the cervix or upper vagina (only to detect more heavily colonized women) showed a strong association with preterm delivery. Likewise, there was a strong evidence of association in case-control studies (Table 1; Supplementary Figures 5 and 6).
3. Exposure classification (prior antibiotic use): higher point estimate in both cohort and case-control studies, but no evidence of association in cohort studies that excluded women using antibiotics. Only 4 cohort studies were included in sensitivity analysis, reducing the power to detect the association (Table 1; Supplementary Figures 7 and 8).
4. Exposure classification (sample timing): The point estimate was higher when samples were taken during delivery/labor (RR, 1.43 [95% CI, .97– 2.11]), than when taken in antenatal visits (RR, 1.20 [95% CI, .85–1.68]), but confidence intervals were overlapping for both estimates (Table 1 and Figure 4).
5. Outcome classification (GA): Point estimates were similar to initial analysis, in studies that measured GA by LMP, ultrasound, and/or fundal height. In case-control studies, where there might be recruitment bias toward more preterm babies included in the studies, there was still evidence of an association (OR, 1.86 [95% CI, 1.15–2.99]; *P* = .011) (Table 1; Supplementary Figures 9 and 10).
6. Outcome classification (preterm definition): No changes in the association were observed when studies with different thresholds for the definition of preterm were excluded in the sensitivity analysis (Table 1; Supplementary Figures 11 and 12).

## DISCUSSION

There is some evidence, from this comprehensive review, that GBS is associated with preterm birth. There is a consistent increase in risk of preterm birth in women with maternal GBS colonization, which is stronger in case-control studies compared to cohort or cross-sectional studies. In addition, where there is evidence of ascending infection with maternal GBS bacteriuria, the association with preterm birth is stronger, which is biologically plausible. Our findings are potentially important, and we have made extensive attempts to consider study design and address specific biases, learning from challenges in previous reviews. However, considerable limitations remain, and these results could still be affected by bias or confounding, as discussed below.

In terms of the data included, we are limited in terms of geographical distribution, with most data from high-income contexts, and the potential sources of bias we were able to assess based on reported sampling strategies, microbiological methods, and gestational age measurement.

Our sensitivity analyses are specifically aimed to address misclassification, but the reduction in power through exclusion of studies likely limited the ability to detect true association. Interestingly, however, in terms of sensitivity of exposure, it may actually be easier to detect an association when less sensitive sampling methods are used. Rectovaginal sampling and broth enrichment increase sensitivity of detection [71] in 40% and 90%, respectively [16], but excluding less sensitive methods did not identify an association. Conversely, using nonselective medium and sampling from the cervix or upper vagina (less sensitive methods that would detect more heavily colonized women), showed a strong association with preterm delivery (RR, 1.65 [95% CI, 1.03–2.64]) for cohort and cross-sectional studies. This is consistent with studies reporting associations in women considered heavily colonized [60] and the association we identified between maternal bacteriuria (which reflects denser colonization) and ascending infection, which was more strongly associated with preterm birth.

Our sensitivity analysis suggested that antibiotic use near or at delivery may also affect findings, and could bias the results toward the null if women with complications are given antibiotics and GBS is thus not detected. In future studies it will be important to take into account the receipt and timing of antibiotics in pregnancy. In terms of the timing of the sample, earlier sampling may increase misclassification due to reacquisition of bacteria, so repeated sampling through pregnancy could be important to test the association.

In terms of outcome measurement, uncertainty in gestational age dating will increase misclassification. This may be nondifferential in cohort and cross-sectional studies, which would bias findings toward the null, but may be differential in case-control studies, with recruitment bias toward more preterm babies included in the study and thus overestimation of the effect. This may account for the differences in findings and some uncertainty in both. Another study found no differences in the effect estimates for GBS colonization and premature delivery when measured by ultrasound or by date of LMP [37], but due to the small number of studies here we could not compare results according to the method used for gestational age dating. We note that the inconsistencies in GA assessment would be even more marked in data from middle-income and especially low-income contexts, where GA measurement is challenging; usually using fundal height and sometimes LMP. Given that three-quarters of preterm births are in sub-Saharan Africa and South Asia, more data, with consistent GA, are crucial for future studies [72].

The results presented here may, however, be subject to confounding (due to factors associated with both maternal GBS colonization and preterm birth). This could change the effect in either direction. Adjustment for confounding factors has increased the effect size in several studies [27, 29, 35, 36, 62], but this may be context specific [16]. It is important that confounding factors are considered, including known risk factors for preterm birth such as low socioeconomic status, black race, low body mass index, previous preterm birth, multiple gestation, short interpregnancy interval, and the use of tobacco or illicit drugs [9], and risk factors for GBS colonization such as age at pregnancy, interpregnancy interval, previous abortions, and level of education [73, 74]. These should be incorporated into multivariable modeling strategies.

To better answer this important research question, more data are needed with optimized and standardized methodologies reported systematically, particularly from low- and middle-income contexts [75]. The optimal study design would be a large, longitudinal prospective study including multiple sites, with accurate exposure and outcome measurement, and repeat sampling at intervals in pregnancy. This should include measurement of gestational age, preferably based on first-trimester ultrasound, samples taken from rectovaginal swabs and isolated in selective enrichment broth (ideally with quantification of GBS colonization), adjustment for use of antibiotics (including timing of receipt of antibiotics during pregnancy), and measurement and adjustment of known risk factors for both preterm delivery and GBS colonization. However, this is challenging to achieve in health systems in low- and middle-income contexts where resources are limited. An alternative approach would be an intervention vaccine-probe study, which would overcome the problems of bias and confounding and could be done as part of a maternal GBS vaccine study.

## CONCLUSIONS

We found some evidence of an association between maternal GBS colonization and preterm birth. Misclassification is likely to reduce the effect size of risk and/or odds ratios, so this may be underestimated. However, results may also be subject to confounding, which could influence the findings in either direction. Current prevention strategies (based on intrapartum antibiotic prophylaxis) are too late to prevent preterm birth associated with GBS colonization. A future maternal GBS vaccine targeted against maternal GBS colonization or the mechanism downstream of colonization leading to preterm birth, and administered during the appropriate timing of pregnancy, could be useful and should be included in any maternal GBS vaccine trial (Table 2).

**Table 2.** Key Findings and Implications

What’s new about this? • This systematic review is the most comprehensive review to date evaluating the association between GBS maternal colonization and preterm delivery, and includes results from 45 studies, split by study design, and 6 sensitivity analyses to assess biases.
What was the main finding? • There is some evidence to suggest that GBS maternal colonization is associated with preterm birth, with cohort/cross-sectional studies suggesting a 20% increased risk and case-control studies an 80% increased risk. Evidence of ascending infection (bacteriuria) carries a higher risk of preterm delivery.
How can the data be improved? • Most of the biases assessed (such as detection of maternal GBS colonization), are likely to bias to the null. However, some biases (eg, recruitment bias for case-control studies), may bias results and overestimate the effect size. Confounding could increase or decrease the effect size, and this may vary in different contexts. Large longitudinal prospective studies that address these biases, confounding, and with accurate exposure and outcome measurement are needed, especially in low- and middle-income contexts.
What does it mean for policy and programs? • Maternal GBS vaccine could reduce preterm birth if associated with maternal GBS colonization, and a vaccine probe study could provide a more definitive answer to this research question.

Abbreviation: GBS, group B *Streptococcus*.

## Supplementary Data

Supplementary materials are available at *Clinical Infectious Diseases* online. Consisting of data provided by the authors to benefit the reader, the posted materials are not copyedited and are the sole responsibility of the authors, so questions or comments should be addressed to the corresponding author.

## Supplementary Material

supplement-material

## Notes

***Author contributions.*** The concept of the estimates and the technical oversight of the series were led by J. E. L. and A. C. S.; F. B. J. performed the database searches, removed duplicates, and screened titles and abstracts for eligibility; M. K. L. and F. B. J. read the full texts, confirmed suitability for inclusion, and extracted data; data analyses were undertaken by F. B. J. and A. C. S.; the first draft of the manuscript and reviews were undertaken by F. B. J., A. C. S., J. E. L., and C. E. R; K. L. D. provided input data for the analyses. The GBS Estimates Expert Advisory Group (C. J. B., L. B., C. C., M. G. G., P. T. H., M. I., K. L. D., S. A. M., S. K. S., S. S., A. S.-t. M., J. V.) contributed to the conceptual process throughout, notably on the disease schema and data inputs. All the authors reviewed and provided input to the manuscript.

***Acknowledgments.*** The authors thank Claudia da Silva for administrative assistance and Alegria Perez for coordinating author signatures.

***Financial support.*** This supplement was supported by a grant to the London School of Hygiene & Tropical Medicine from the Bill & Melinda Gates Foundation (Grant ID: OPP1131158).

***Supplement sponsorship.***  This article appears as part of the supplement “The Burden of Group B *Streptococcus* Worldwide for Pregnant Women, Stillbirths, and Children,” sponsored by the Bill & Melinda Gates Foundation and coordinated by the London School of Hygiene & Tropical Medicine.

***Potential conflicts of interest.*** Many contributors to this supplement have received funding for their research from foundations, especially the Bill & Melinda Gates Foundation, and several from the Wellcome Trust, the Medical Research Council UK, the Thrasher Foundation, the Meningitis Research Foundation, and one individual from the US National Institutes of Health. Members of the Expert Advisory Group received reimbursement for travel expenses to attend working meetings related to this series. A. S.-t. M. works for the Bill & Melinda Gates Foundation. C. J. B. has served as a member of the Presidential Advisory Committee for Seqirus Inc and of the CureVac Inc Scientific Advisory Committee, as well as undertaken consultancy work for Pfizer Inc. C. C. has received institutional compensation from Novartis for conducting GBS studies. P. T. H. has been a consultant to Novartis and Pfizer on GBS vaccines but received no funding for these activities. M. I. has undertaken sponsored research from Pfizer on pneumococcal disease in adults and from Belpharma Eumedica (Belgium) on temocillin antimicrobial susceptibility in Enterobacteriaceae. K. L. D. has received funding by the Bill & Melinda Gates Foundation to work on research on GBS serocorrelates of protection to inform vaccine trials, and travel expenses from Pfizer to attend a meeting on an investigator-led project on GBS. S. A. M. has collaborated on GBS grants funded by GlaxoSmithKline and by Pfizer and received personal fees for being member of its advisory committee; he has also collaborated on a GBS grant funded by Minervax. All other authors report no potential conflicts of interest. All authors have submitted the ICMJE Form for Disclosure of Potential Conflicts of Interest. Conflicts that the editors consider relevant to the content of the manuscript have been disclosed.
