# Supplementary material for: Preterm Birth Associated With Group B Streptococcus Maternal Colonization Worldwide: Systematic Review and Meta-analyses
Source: Clin Infect Dis. 2017 Nov 6;65(Suppl 2):S133–42. doi: 10.1093/cid/cix661 (PMC5850429; doi:10.1093/cid/cix661)

The burden of Group B *Streptococcus* worldwide for pregnant women, stillbirths and children

Paper 5: Preterm birth associated with Group B *Streptococcus* maternal colonization worldwide: systematic review and meta-analyses

Supplementary information

Contents

The burden of Group B *Streptococcus* worldwide for pregnant women, stillbirths and children 1

Paper 5: Preterm birth associated with Group B *Streptococcus* maternal colonization worldwide: systematic review and meta-analyses 1

Supplementary information 1

Supplementary Table S1: search terms 3

Supplementary Table S2: inclusion and exclusion criteria 5

Supplementary Table S3: Study characteristics and data 6

Supplementary Figure S1: Meta-analysis of cohort studies with GBS colonization detected from urine samples by time of sampling. 19

Supplementary Figure S2: Meta-analysis of case control studies with GBS colonization detected from urine samples. 20

Supplementary Figure S3: Meta-analysis of cohort and cross sectional studies for studies using recto-vaginal sampling and broth selective enrichment for isolation of GBS by time of sampling 21

Supplementary Figure S4: Meta-analysis of case-control studies for studies using recto-vaginal sampling and broth selective enrichment for isolation of GBS 22

Supplementary Figure S5: Meta-analysis of cohort and cross sectional studies using nonselective laboratory methods and cervical and upper vaginal sampling by time of sampling 23

Supplementary Figure S6: Meta-analysis of case-control studies using nonselective laboratory methods and cervical and upper vaginal sampling 24

Supplementary Figure S7: Meta-analysis of cohort and cross sectional studies where mothers reported not using antibiotics (AB) during pregnancy or at least one week before the culture sample was taken, by time of sampling 25

Supplementary Figure S8: Meta-analysis of case-control studies where mothers reported not using antibiotics (AB) during pregnancy or at least one week before the culture sample was taken 26

Supplementary Figure S9: Meta-analysis of cohort and cross sectional studies for studies measuring GA by LMP, ultrasound and/or fundal height 27

Supplementary Figure S10: Meta-analysis of case-control studies for studies for studies measuring GA by LMP, ultrasound and/or fundal height 28

Supplementary Figure S11: Meta-analysis without cohort and cross-sectional studies using different thresholds for the definition of preterm (or not specifying the definition) by time of sampling 29

Supplementary Figure 12: Meta-analysis without case-control studies using different thresholds for the definition of preterm (or not specifying the definition) 30

Supplementary Table S1: search terms

("infant, premature"[MeSH Terms] OR ("infant"[All Fields] AND "premature"[All Fields]) OR "premature infant"[All Fields] OR "prematurity"[All Fields]) OR ("infant, premature"[MeSH Terms] OR ("infant"[All Fields] AND "premature"[All Fields]) OR "premature infant"[All Fields] OR ("prematurity"[All Fields] AND "infant"[All Fields])) OR (premature [All Fields] AND ("infant, newborn"[MeSH Terms] OR ("infant"[All Fields] AND "newborn"[All Fields]) OR "newborn infant" [All Fields] OR "neonate"[All Fields])) OR ("infant, premature"[MeSH Terms] OR ("infant"[All Fields] AND "premature"[All Fields]) OR "premature infant"[All Fields] OR ("premature"[All Fields] AND "baby"[All Fields]) OR "premature baby"[All Fields]) OR ("premature birth"[MeSH Terms] OR ("premature"[All Fields] AND "birth"[All Fields]) OR "premature birth"[All Fields]) OR ("premature birth"[MeSH Terms] OR ("premature"[All Fields] AND "birth"[All Fields]) OR "premature birth"[All Fields] OR ("premature"[All Fields] AND "childbirth"[All Fields]) OR "premature childbirth"[All Fields]) OR (premature[All Fields] AND ("infant, newborn"[MeSH Terms] OR ("infant"[All Fields] AND "newborn"[All Fields]) OR "newborn infant"[All Fields] OR "newborn"[All Fields]))

OR prematuritas[All Fields] OR ("premature labour"[All Fields] OR "obstetric labor, premature"[MeSH Terms] OR ("obstetric"[All Fields] AND "labor"[All Fields] AND "premature"[All Fields]) OR "premature obstetric labor"[All Fields] OR ("premature"[All Fields] AND "labor"[All Fields]) OR "premature labor"[All Fields]) OR ("obstetric labor, premature"[MeSH Terms] OR ("obstetric"[All Fields] AND "labor"[All Fields] AND "premature"[All Fields]) OR "premature obstetric labor"[All Fields] OR ("labor"[All Fields] AND "premature"[All Fields]) OR "labor, premature"[All Fields]) OR ("obstetric labor, premature"[MeSH Terms] OR ("obstetric"[All Fields] AND "labor"[All Fields] AND "premature"[All Fields]) OR "premature obstetric labor"[All Fields] OR ("labor"[All Fields] AND "premature"[All Fields]) OR "obstetric labor, premature"[All Fields])

OR ("premature birth"[MeSH Terms] OR ("premature"[All Fields] AND "birth"[All Fields]) OR "premature birth"[All Fields] OR ("premature"[All Fields] AND "delivery"[All Fields]) OR "premature delivery"[All Fields]) OR ("infant, premature"[MeSH Terms] OR ("infant"[All Fields] AND "premature"[All Fields]) OR "premature infant"[All Fields] OR ("premature"[All Fields] AND "babies"[All Fields]) OR "premature babies"[All Fields]) OR ("infant, premature"[MeSH Terms] OR ("infant"[All Fields] AND "premature"[All Fields]) OR "premature infant"[All Fields] OR ("preterm"[All Fields] AND "infant"[All Fields]) OR "preterm infant"[All Fields]) OR ("premature birth"[MeSH Terms] OR ("premature"[All Fields] AND "birth"[All Fields]) OR "premature birth"[All Fields] OR ("preterm"[All Fields] AND "delivery"[All Fields]) OR "preterm delivery"[All Fields])

OR ("obstetric labor, premature"[MeSH Terms] OR ("obstetric"[All Fields] AND "labor"[All Fields] AND "premature"[All Fields]) OR "premature obstetric labor"[All Fields] OR ("preterm"[All Fields] AND "labor"[All Fields]) OR "preterm labor"[All Fields]) OR (preterm[All Fields] AND ("infant, newborn"[MeSH Terms] OR ("infant"[All Fields] AND "newborn"[All Fields]) OR "newborn infant"[All Fields] OR "neonate"[All Fields])) OR ("premature birth"[MeSH Terms] OR ("premature"[All Fields] AND "birth"[All Fields]) OR "premature birth"[All Fields] OR ("preterm"[All Fields] AND "birth"[All Fields]) OR "preterm birth"[All Fields]) OR ("infant, premature"[MeSH Terms] OR ("infant"[All Fields] AND "premature"[All Fields]) OR "premature infant"[All Fields] OR ("preterm"[All Fields] AND "baby"[All Fields]) OR "preterm baby"[All Fields]) OR (preterm[All Fields] AND ("infant"[MeSH Terms] OR "infant"[All Fields] OR "babies"[All Fields])) OR (pre-term[All Fields] AND ("infant, newborn"[MeSH Terms] OR ("infant"[All Fields] AND "newborn"[All Fields]) OR "newborn

infant"[All Fields] OR "baby"[All Fields] OR "infant"[MeSH Terms] OR "infant"[All Fields])) OR (pre-term[All Fields] AND ("parturition"[MeSH Terms] OR "parturition"[All Fields] OR "birth"[All Fields])) OR (pre-term[All Fields] AND ("infant"[MeSH Terms] OR "infant"[All Fields] OR "babies"[All Fields])) OR (pre-mature[All Fields] AND ("delivery, obstetric"[MeSH Terms] OR ("delivery"[All Fields] AND "obstetric"[All Fields]) OR "obstetric delivery"[All Fields] OR "delivery"[All Fields])) OR (pre-mature[All Fields] AND ("infant, newborn"[MeSH Terms] OR ("infant"[All Fields] AND "newborn"[All Fields]) OR "newborn infant"[All Fields] OR "baby"[All Fields] OR "infant"[MeSH Terms] OR "infant"[All Fields]))

(Taken from Blencowe et al. 2012)

AND

("Streptococcus" [All Fields] OR "Streptococcal" [All Fields] OR "Streptococci" [All Fields]) AND (("Group" AND "B") or "Agalactiae") OR "Streptococcus Agalactiae" [MeSH Terms]

Limit: humans

Supplementary Table S2: inclusion and exclusion criteria

	Inclusion criteria	Exclusion criteria
**Population**	Number of preterm and term births in both GBS and non-GBS carriers	Non appropriate comparison group
**Laboratory/definitions**	Recto-vaginal swab (or rectal or vaginal), vaginal, cervical<br>Urine, chorioamnion or mixed	Preterm labor not properly defined
**Search**	No language limits<br>No date limits	Conference abstracts where it was not possible to obtain further information

Supplementary Table S3: Study characteristics and data

Study	Year of publication	Country	Study design	Time of screening	Definition of outcome (preterm birth/labor)	Site of sample taken	Lab methods	Gestational age measured	Exclusion of women who had antibiotics	Sample size	Risk ratio/ Odds ratio	95% confidence interval
Aali	2007	Iran	Case-control	labor/delivery	Four uterine contractions in 20 minutes time period with cervical dilatation >1cm and cervical effacement of ≥80% before 37 weeks' gestation	vaginal	blood and chocolate agar plates	date of last menstrual period or ultrasonographic report in the first half of pregnancy	women in current antibiotic usage	206	1.89	0.71-5.01
Agger	2014	USA	Cohort	11.5 (± 3.7) weeks' gestation	delivery <37 weeks' gestation	cervical	not specified			676	0.37	0.15-0.90
Allen	1999	Canada	Cohort (retrospective)	early third trimester (26-28 weeks gestational age)	delivery <37 weeks' gestation	vaginal or vaginal-anorectal	GBS broth (Lim broth/selective media)			986	2.10	1.38-3.20
Anderson	2007	USA	Cohort (retrospective)	before 20 week's gestation	delivery <37 weeks' gestation	urine cultures	sheep blood agar, macconkey agar and GBS agar plates			243	1.79	1.01-3.17
Choi	2012	Korea	Case-control	at delivery	not specified	vaginal, anorectum, and urethral	Todd-Hewitt broth and new Granada plate			160	0.45	0.16-1.21

Study	Year of publication	Country	Study design	Time of screening	Definition of outcome (preterm birth/labor)	Site of sample taken	Lab methods	Gestational age measured	Exclusion of women who had antibiotics	Sample size	Risk ratio/ Odds ratio	95% confidence interval
						orifice swabs						
Citernesi	1996	Italy	Cross-sectional	at delivery	delivery <37 weeks' gestation	vaginal	blood agar plates			4672	1.33	0.83-2.13
Dahan-Saal	2011	France	Cohort (retrospective)	not specified	delivery <37 weeks' gestation	vaginal (positive women also urine and newborn)	Polyvitex1 and gels to ANC			8733	1.13	0.98-1.30
Daskalakis	2006	Greece	Cohort	22 and 25 weeks' gestation	delivery <37 weeks' gestation	vaginal	blood agar plates with gentamicin and nalidixic acid	date of last menstrual period or a 1st trimester scan if there was a discrepancy of more than a weeks	women who received an antibiotic effective against BV or GBS at any gestational age following the screening	1197	0.43	0.19-0.96
Discacciati	2011	Brazil	Case-control	at labor	One uterine contraction every 5-8 minutes with cervical dilatation of >2cm and/or	vaginal	blood agar plates		women who used antibiotics during the two preceding weeks	82	5.33	0.57-49.97

Study	Year of publication	Country	Study design	Time of screening	Definition of outcome (preterm birth/labor)	Site of sample taken	Lab methods	Gestational age measured	Exclusion of women who had antibiotics	Sample size	Risk ratio/ Odds ratio	95% confidence interval
					cervical effacement of ≥50% before 37 weeks' gestation							
Feikin	2001	Denmark	Cohort	at enrollment ≤ 24 weeks' gestation	delivery <37 weeks' gestation	cervical and vaginal	blood agar plates	date of the last menstrual period and ultrasonographic measurements at 18 weeks gestation were used to confirm gestational age		2846	0.97	0.47-1.98
Feikin (continued)	2001	Denmark	Cohort	at delivery	delivery <37 weeks' gestation	cervical and vaginal	blood agar plates	date of the last menstrual period and ultrasonographic measurements at 18 weeks gestation were used to confirm gestational age		384	1.72	1.04-2.83

Study	Year of publication	Country	Study design	Time of screening	Definition of outcome (preterm birth/labor)	Site of sample taken	Lab methods	Gestational age measured	Exclusion of women who had antibiotics	Sample size	Risk ratio/ Odds ratio	95% confidence interval
Feikin (continued)	2001	Denmark	Case-control	Controls had specimens taken during routine visits in prenatal clinic; cases specimens taken during labor	delivery <37 weeks' gestation	cervical and vaginal	blood agar plates	date of the last menstrual period and ultrasonographic measurements at 18 weeks gestation were used to confirm gestational age		308	1.91	0.88-4.15
Garland	2000	Australia	Cohort (retrospective)	at week 28 or 32 of gestation	delivery <37 weeks' gestation	vaginal	Todd-Hewitt broth with crystal violet, colistin, and nalidixic acid	Both by ultrasound and by dates		1120	0.53	0.37-0.75
Gerards	1982	Netherlands	Cohort	before 20th week, at 28th and 34th week's gestation and at delivery	not specified	vaginal, cervical and rectal	sheep blood agar plate and selective broth medium of Pike			161	0.62	0.26-1.50
Gojnic	2005	Serbia and Montenegro	Case-control	at delivery	delivery between 24 to 37 weeks of gestation	cervical	not specified			232	2.43	0.44-13.54

**Study**	**Year of publication**	**Country**	**Study design**	**Time of screening**	**Definition of outcome (preterm birth/labor)**	**Site of sample taken**	**Lab methods**	**Gestational age measured**	**Exclusion of women who had antibiotics**	**Sample size**	**Risk ratio/ Odds ratio**	**95% confidence interval**
Hakansson	2008	Sweden	Cross-sectional	at delivery	delivery <37 weeks' gestation	vaginal and rectal	Todd-Hewitt with nalidixic acid and gentamicin			1507	0.63	0.37-1.07
Hassanzadeh	2011	Iran	Cross-sectional	at delivery	delivery <37 weeks' gestation	vaginal-rectal	Todd-Hewitt broth with gentamicin and nalidixic acid			310	1.55	0.34-7.07
Hastings	1986	UK	Cohort	at booking, 28 and 36 weeks' gestation and during labor	delivery <37 weeks' gestation	vaginal and rectal	blood agar and Islam's starch serum agar with with nalidixic acid and gentamicin sulphate, Todd Hewitt broth			1059	1.01	0.60-1.68
Hillier	1991	USA	Case-control	at delivery	delivery at ≤34 completed weeks	chorioamnion cultures and placenta histologic samples	not specified	date of last menstrual period, fundal height, ultrasonography and an evaluation of the newborn with a standardized Ballard examination	women who had received antibiotics in the previous week	268	3.92	1.02-15.13

Study	Year of publication	Country	Study design	Time of screening	Definition of outcome (preterm birth/labor)	Site of sample taken	Lab methods	Gestational age measured	Exclusion of women who had antibiotics	Sample size	Risk ratio/ Odds ratio	95% confidence interval
Jones	2006	UK	Cohort	between 34 weeks to full term	delivery <37 weeks' gestation	vaginal-rectal	Todd-Hewitt broth with gentamicin and nalidixic acid and NNA plate with neomycin and nalidixic acid			167	0.00	
Joshi	1987	Canada	Cross-sectional	at delivery	delivery at or before 37 weeks gestation	vaginal	blood agar plates			3078	2.49	1.62-3.83
Kessous	2012	Israel	Cohort (retrospective)	not specified	delivery <37 weeks' gestation	vaginal	not specified			216132	0.76	0.66-0.88
Kessous(continued)	2012	Israel	Cohort (retrospective)	not specified	delivery <37 weeks' gestation	urine cultures	not specified			215393	1.94	1.76-2.14
Khalil	2015	Denmark	Cohort (retrospective)	during pregnancy	not specified	urine cultures	not specified			13417	1.66	1.41-1.97
Kim	2015	Korea	Cohort	last trimester	not specified	vaginal and rectal	Todd-Hewitt broth and CHROMagar StrepB agar (CHROM-B)			107	1.38	0.77-2.45

Study	Year of publication	Country	Study design	Time of screening	Definition of outcome (preterm birth/labor)	Site of sample taken	Lab methods	Gestational age measured	Exclusion of women who had antibiotics	Sample size	Risk ratio/ Odds ratio	95% confidence interval
Kovachev	2003	Bulgaria	Cohort	24 weeks' gestation	not specified	cervical and vaginal	blood agar with gentamicin and nalidixic		women who used antibiotics therapy within the past two weeks	110	10.22	2.81-37.16
Lamont	1986	UK	Case-control	at admission for preterm labor or 24 h before elective delivery (controls)	At least two uterine contractions in 10 minutes (gestation weeks not specified)	cervical, vaginal and rectal	neomycin blood agar and enrichment medium with gentamicin and nalidixic acid			98	0.00	
LeDoare	unpublished data	Gambia	Cohort	at delivery	delivery <37 weeks' gestation	vaginal-rectal	Todd-Hewitt enrichment and agar plating CCNA	Ballard score		750	1.07	0.88-1.29
Martius	1988	USA	Case-control	at delivery	Two or more uterine contractions within 10 minutes, lasting longer than 2 hours before 37 weeks' gestation	vaginal and cervical	sheep blood agar and Todd-Hewitt broth with gentamicin and naladixic acid	date of last menstrual period, fundal height, and ultrasonography when available	women who used within two weeks of enrollment	212	1.82	0.84-3.97

Study	Year of publication	Country	Study design	Time of screening	Definition of outcome (preterm birth/labor)	Site of sample taken	Lab methods	Gestational age measured	Exclusion of women who had antibiotics	Sample size	Risk ratio/ Odds ratio	95% confidence interval
Matorras	1989	Spain	Cohort	samples from range of 17th to 42nd weeks' gestation	delivery <37 completed weeks' gestation	vaginal and rectal	blood agar with nalidixic acid and Todd-Hewitt broth with blood and nalidixic acid	date of last menstrual period		1011	0.91	0.58-1.43
Mc Kenzie	1994	UK	Cohort	28 weeks	delivery <37 weeks' gestation	urine samples	blood agar plates	Menstrual data and corrected if early ultrasound showed a discrepancy of greater than one week		1866	0.31	0.04-2.16
Mikhova	2007	Bulgaria	Case-control	not specified	delivery <34 weeks	vaginal	not specified		women who had antibiotic treatment previous to the sample taking	88	3.29	0.60-17.95

Study	Year of publication	Country	Study design	Time of screening	Definition of outcome (preterm birth/labor)	Site of sample taken	Lab methods	Gestational age measured	Exclusion of women who had antibiotics	Sample size	Risk ratio/ Odds ratio	95% confidence interval
Minkoff	1984	USA	Cohort	first prenatal visit (13.8 +/- 3.6 weeks' gestation)	More than one contraction every 8 minutes and cervical effacement >75% or >1cm dilatation before 37 weeks' gestation	vaginal	modified Thayer-Martin agar , chocolate agar, tryptic soy agar containing sheep blood, MacConkey agar, and bile esculin azide agar		women were not given antibiotics after cultures were obtained	218	1.84	0.86-3.94
Moller	1984	Denmark	Cohort	at least once between 12 and 38 weeks' gestation	delivery before the end of week 37 of gestation	urine samples	not specified			2745	2.52	1.55-4.08
Persson	1986	Sweden	Case-control	at delivery	delivery <37 weeks' gestation	urethral, rectal and urine	Todd-Hewitt broth with gentamicin and nalidixic acid			366	1.21	0.49-2.99
Petersen	2014	Denmark	Cohort (retrospective)	during pregnancy	delivery <37 weeks' gestation	urine samples and lower vagina swabs	blood agar resistance plate and a chromID CPS plate specific for GBS			35175	1.81	1.57-2.10

Study	Year of publication	Country	Study design	Time of screening	Definition of outcome (preterm birth/labor)	Site of sample taken	Lab methods	Gestational age measured	Exclusion of women who had antibiotics	Sample size	Risk ratio/ Odds ratio	95% confidence interval
Regan	1981	USA	Cohort	at delivery	delivery <32 weeks' gestation	cervical	not specified	date of last menstrual period, when in doubt Usher score of physical examination criteria of the infant during first 24 hours of life		6706	4.11	2.88-5.87
Regan	1996	USA	Cohort	at 23 to 26 weeks' gestation	delivery <37 completed weeks' gestation	vaginal, endocervical swab	selective broth media containing gentamicin and nalidixic acid	date of last menstrual period, and other clinical evidence such as uterine size, detection of fetal heart tones and ultrasonography when done	results for women who were not administered effective antibiotics	10385	1.04	0.91-1.20
Schwab	2016	Indonesia	Cohort	second trimester of pregnancy	delivery <37 weeks' gestation	vaginal swabs	not specified	Self-reported last menstrual period		62	2.22	1.24-3.98

Study	Year of publication	Country	Study design	Time of screening	Definition of outcome (preterm birth/labor)	Site of sample taken	Lab methods	Gestational age measured	Exclusion of women who had antibiotics	Sample size	Risk ratio/ Odds ratio	95% confidence interval
Seale	2016	Kenya	Cohort	at delivery	delivery ≥32 <37 weeks' gestation	recto-vaginal swabs	Amies transport medium with charcoal, enrichment Lim broth and subculture onto blood agar	last menstrual period when available (fundal height if not)		7408	0.89	0.80-0.99
Seoud	2010	Lebanon	Cross-sectional	at delivery	delivery ≤36 weeks' gestation	vaginal and rectal	SBA plate			774	0.93	0.51-1.68
Seyyed	2013	Iran	Case-control	at admission to hospital for labor	Four in 20 minutes or eight in 60 minutes uterine contractions and progressive change in cervix or ≥80% cervical effacement between 20-37 weeks' gestation	vaginal and rectal	Todd-Hewitt broth with gentamicin and nalidixic acid		women who used antibiotics during two preceding weeks	329	2.53	1.55-4.13

Study	Year of publication	Country	Study design	Time of screening	Definition of outcome (preterm birth/labor)	Site of sample taken	Lab methods	Gestational age measured	Exclusion of women who had antibiotics	Sample size	Risk ratio/ Odds ratio	95% confidence interval
Sweet	1987	USA	Cohort	initial prenatal visit and repeated at 30 to 34 weeks' gestation	not specified	vaginal	trypicase soy agar plate with sheep blood (TSB) and NPC broth			3028	1.2	0.83-1.74
Thomsen	1987	Denmar k	Cohort	between 27 and 31 weeks' gestation	delivery before end of week 37 of gestation	urine cultures	not specified		women in a trial, results for GBS positive women given placebo	4085	7.98	5.00-12.74
Tsolia	2003	Greece	Cohort	during follow-up exam (≥ 35 weeks) or at labor	delivery <37 weeks' gestation	vaginal and rectal	Todd-Hewitt broth with gentamicin and nalidixic acid			1014	1.17	0.54-2.54
Tsui	2002	Hong Kong	Cohort	at first or second trimester	delivery ≤ 37 weeks' gestation	vaginal and rectal	Todd-Hewitt broth with gentamicin and nalidixic acid			952	1.09	0.48-2.48
White	1984	UK	Cohort	antenatal	labor <37 weeks' gestation	urine cultures	not specified			8083	1.49	0.81-2.73

Study	Year of publication	Country	Study design	Time of screening	Definition of outcome (preterm birth/labor)	Site of sample taken	Lab methods	Gestational age measured	Exclusion of women who had antibiotics	Sample size	Risk ratio/ Odds ratio	95% confidence interval
Wilk	2003	Poland	Cross-sectional	at delivery	delivery before the end of 37th week	cervical	sheep blood agar, MacConkey agar and Chapman agar, brain heart infusion broth			656	1.85	1.01-3.14

Supplementary Figure S1: Meta-analysis of cohort studies with GBS colonization detected from urine samples by time of sampling.

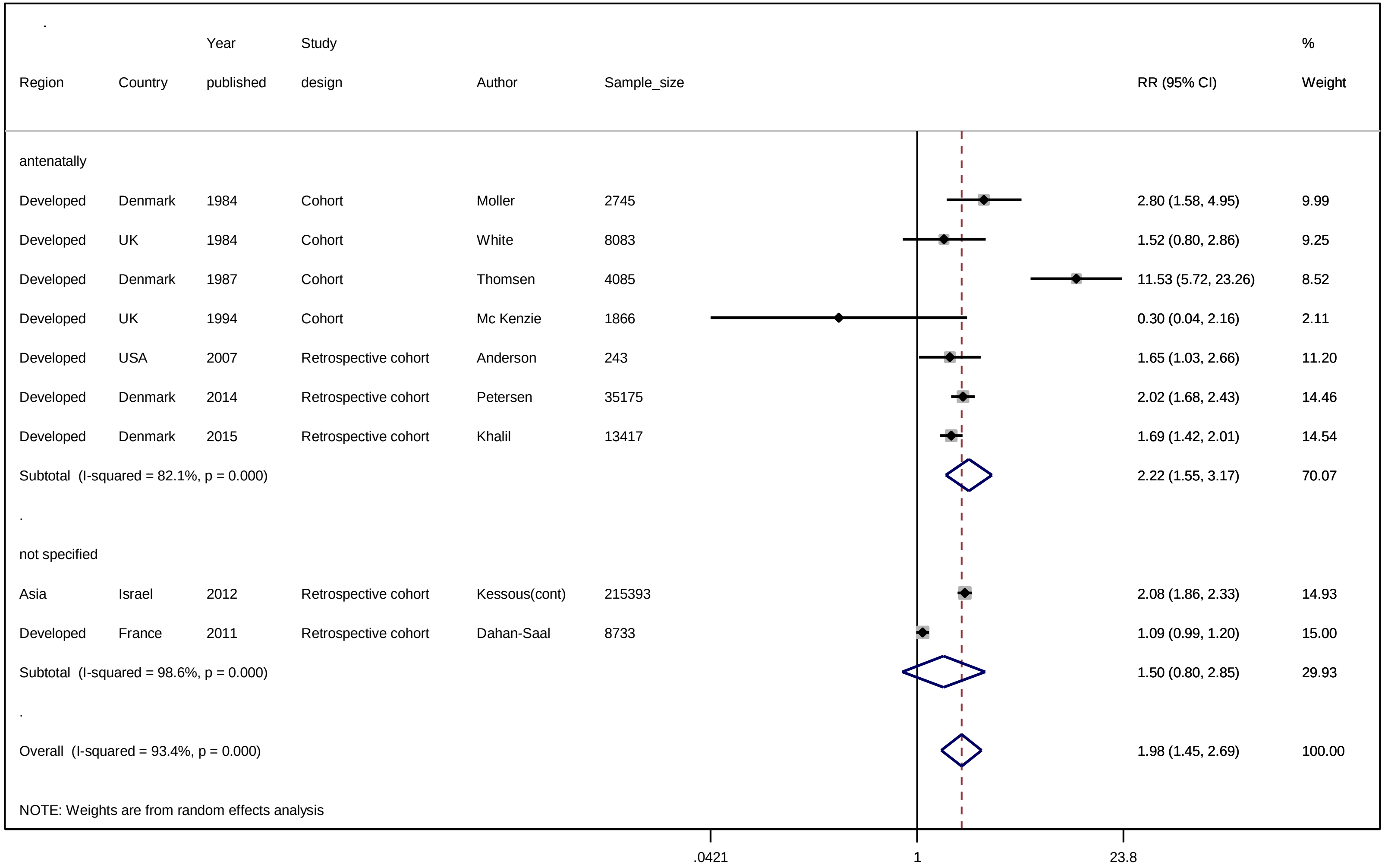

Supplementary Figure S2: Meta-analysis of case control studies with GBS colonization detected from urine samples.

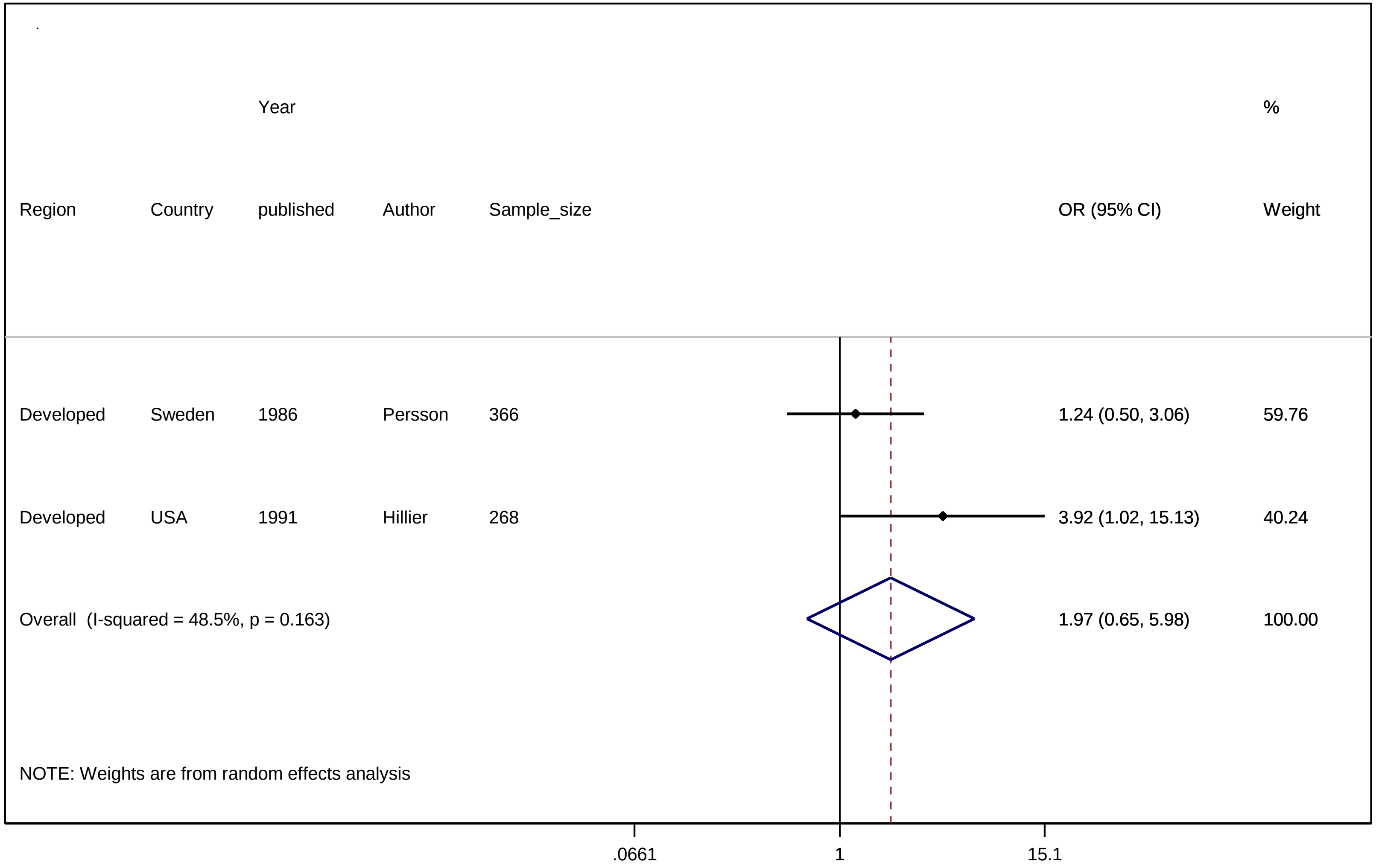

Supplementary Figure S3: Meta-analysis of cohort and cross sectional studies for studies using recto-vaginal sampling and broth selective enrichment for isolation of GBS by time of sampling

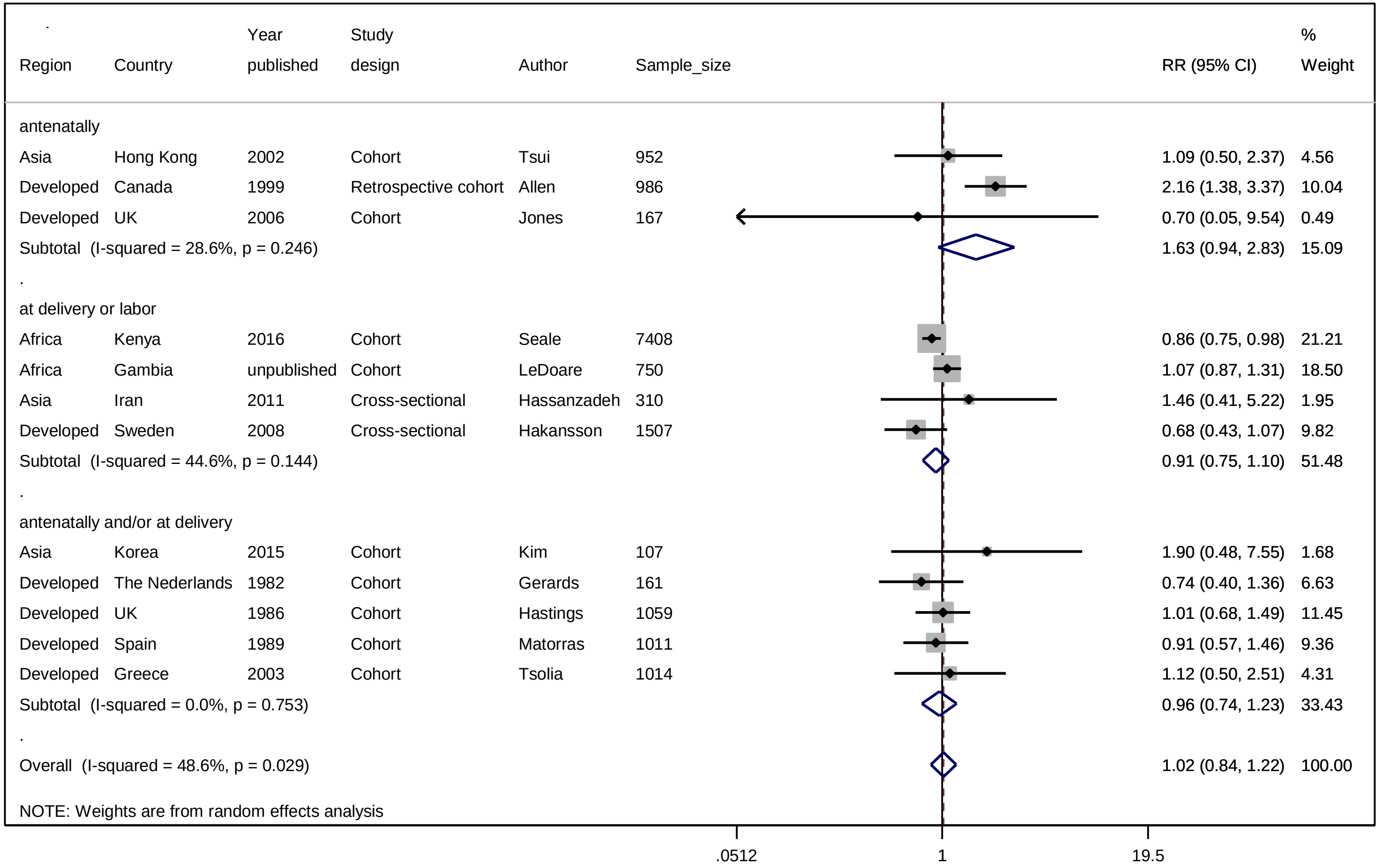

Supplementary Figure S4: Meta-analysis of case-control studies for studies using recto-vaginal sampling and broth selective enrichment for isolation of GBS

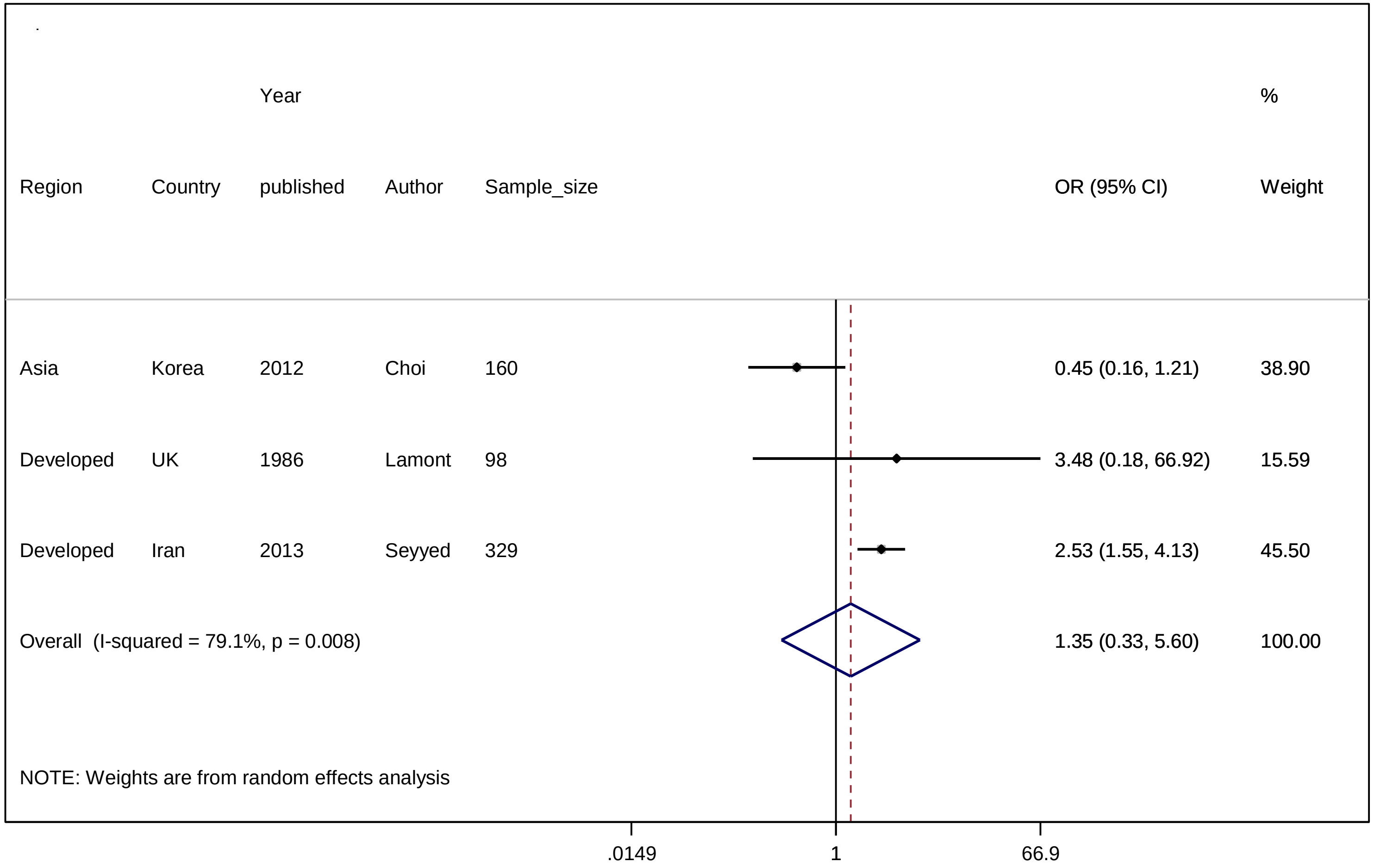

Supplementary Figure S5: Meta-analysis of cohort and cross sectional studies using nonselective laboratory methods and cervical and upper vaginal sampling by time of sampling

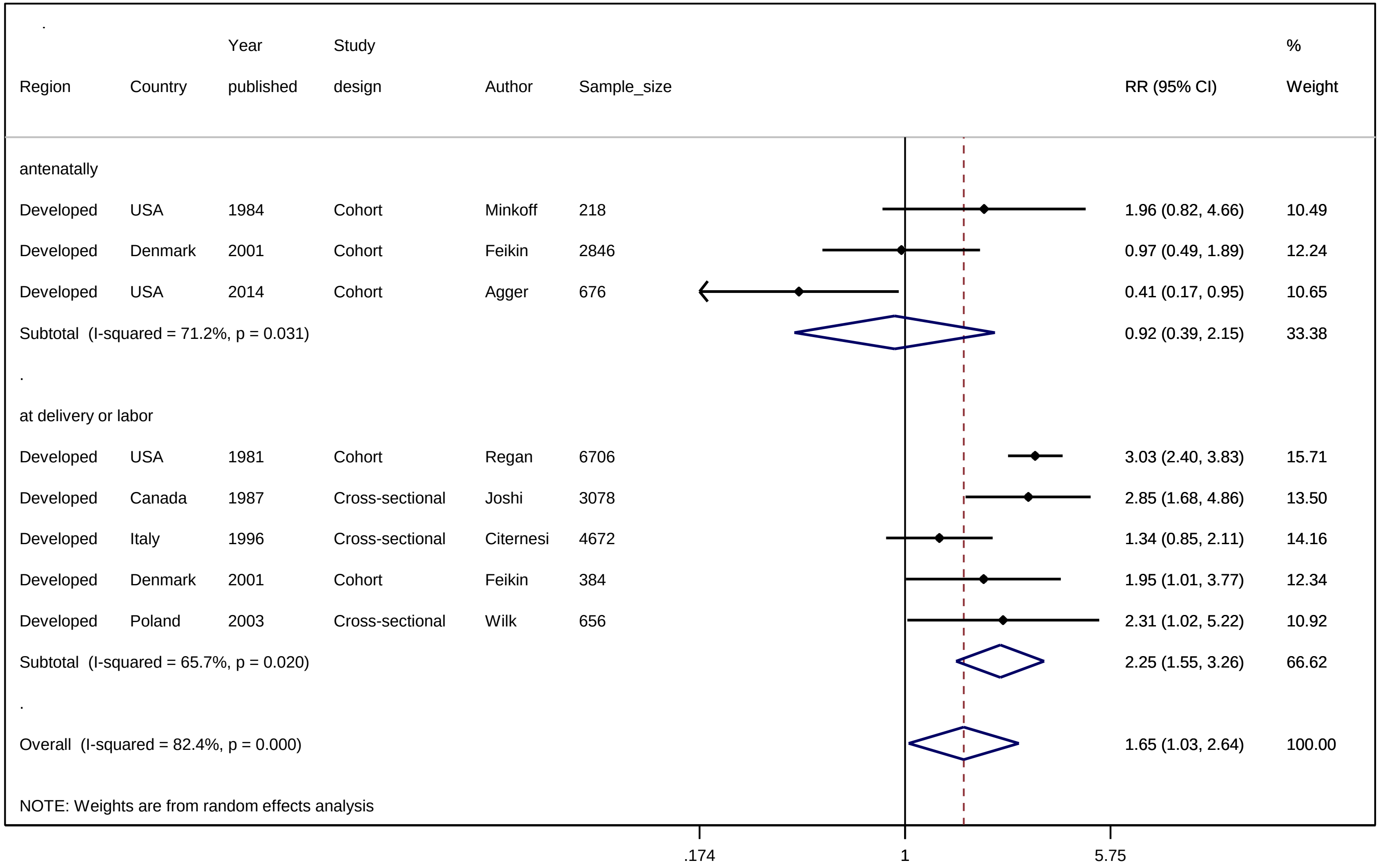

Supplementary Figure S6: Meta-analysis of case-control studies using nonselective laboratory methods and cervical and upper vaginal sampling

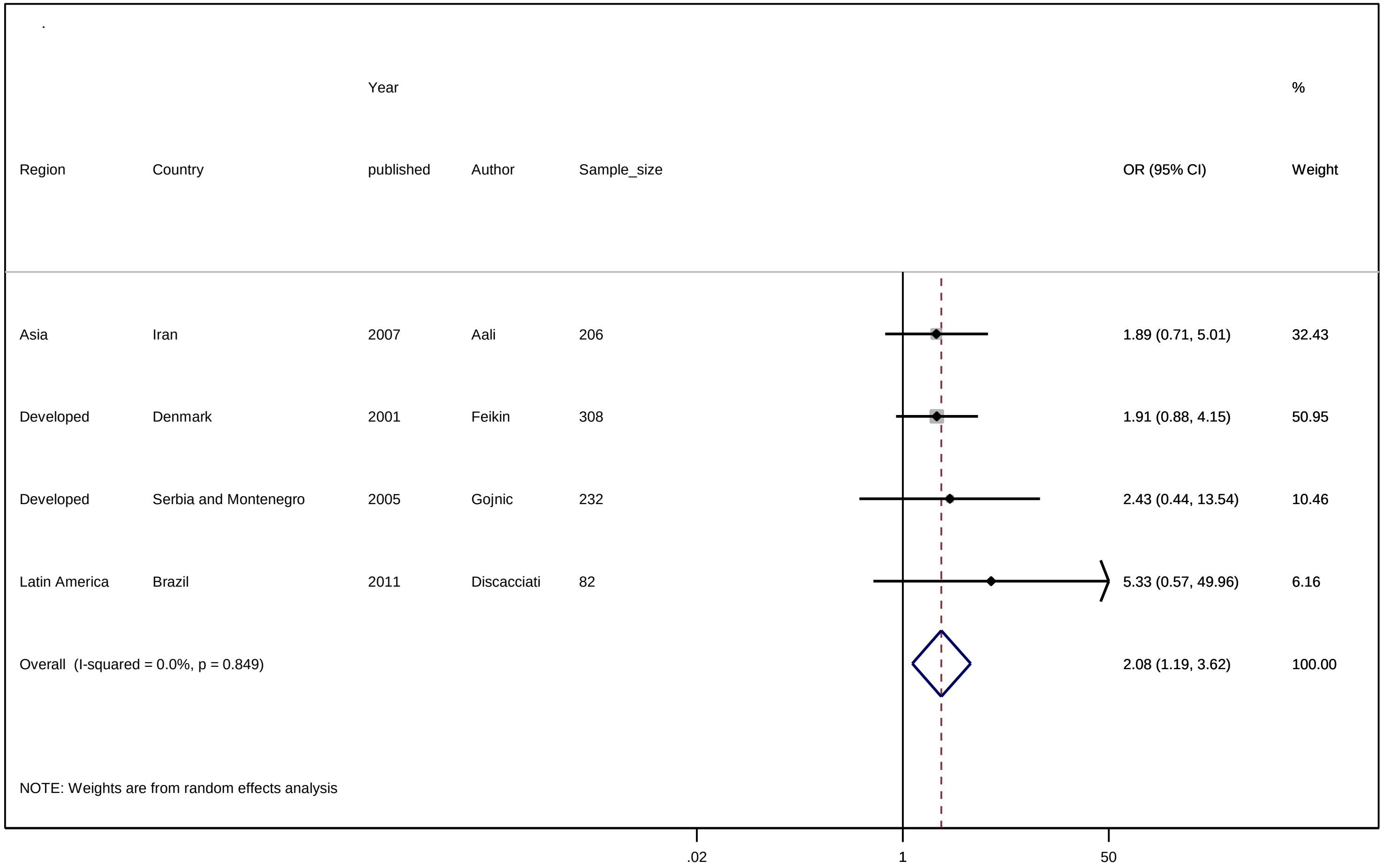

Supplementary Figure S7: Meta-analysis of cohort and cross sectional studies where mothers reported not using antibiotics (AB) during pregnancy or at least one week before the culture sample was taken, by time of sampling

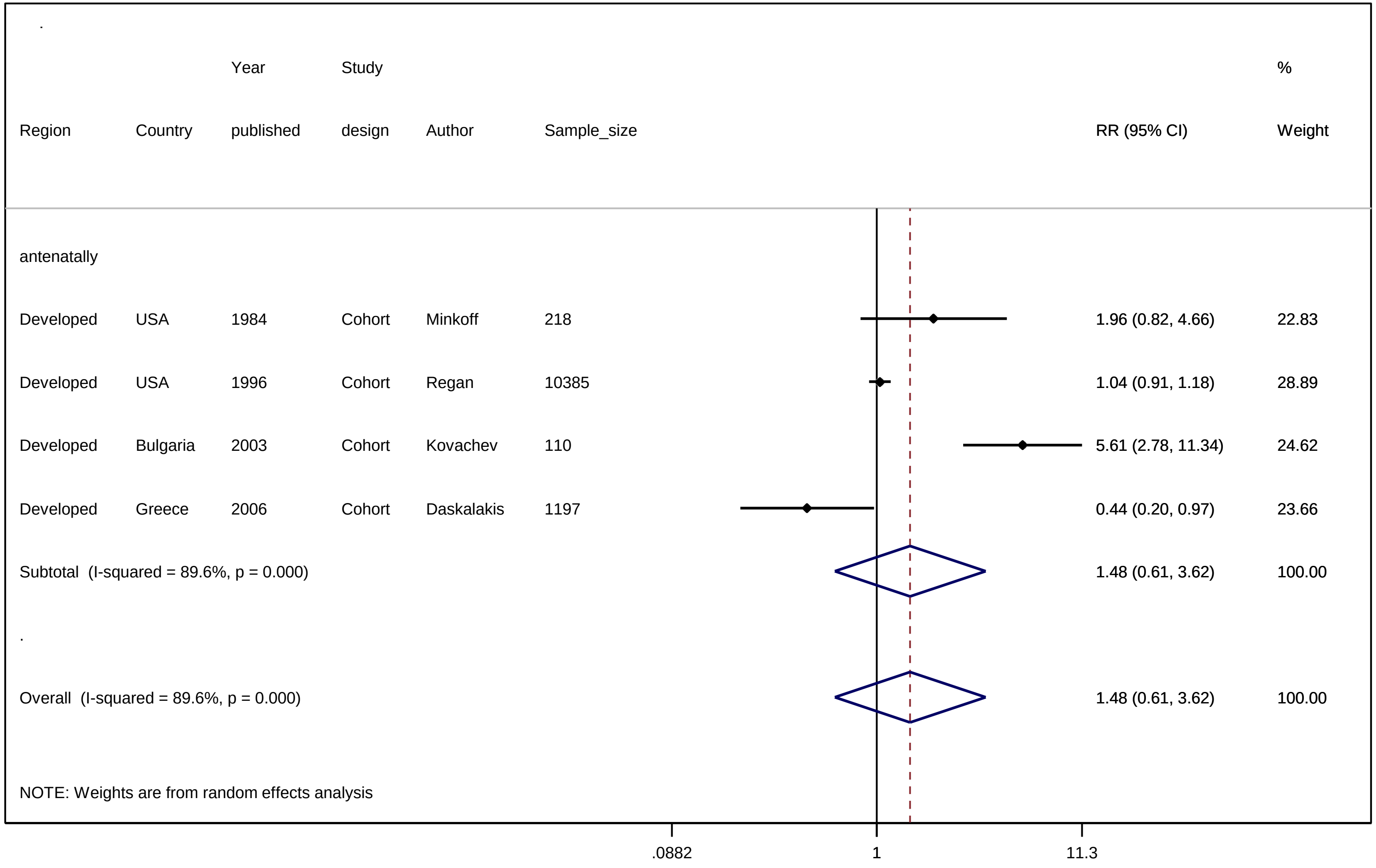

Supplementary Figure S8: Meta-analysis of case-control studies where mothers reported not using antibiotics (AB) during pregnancy or at least one week before the culture sample was taken

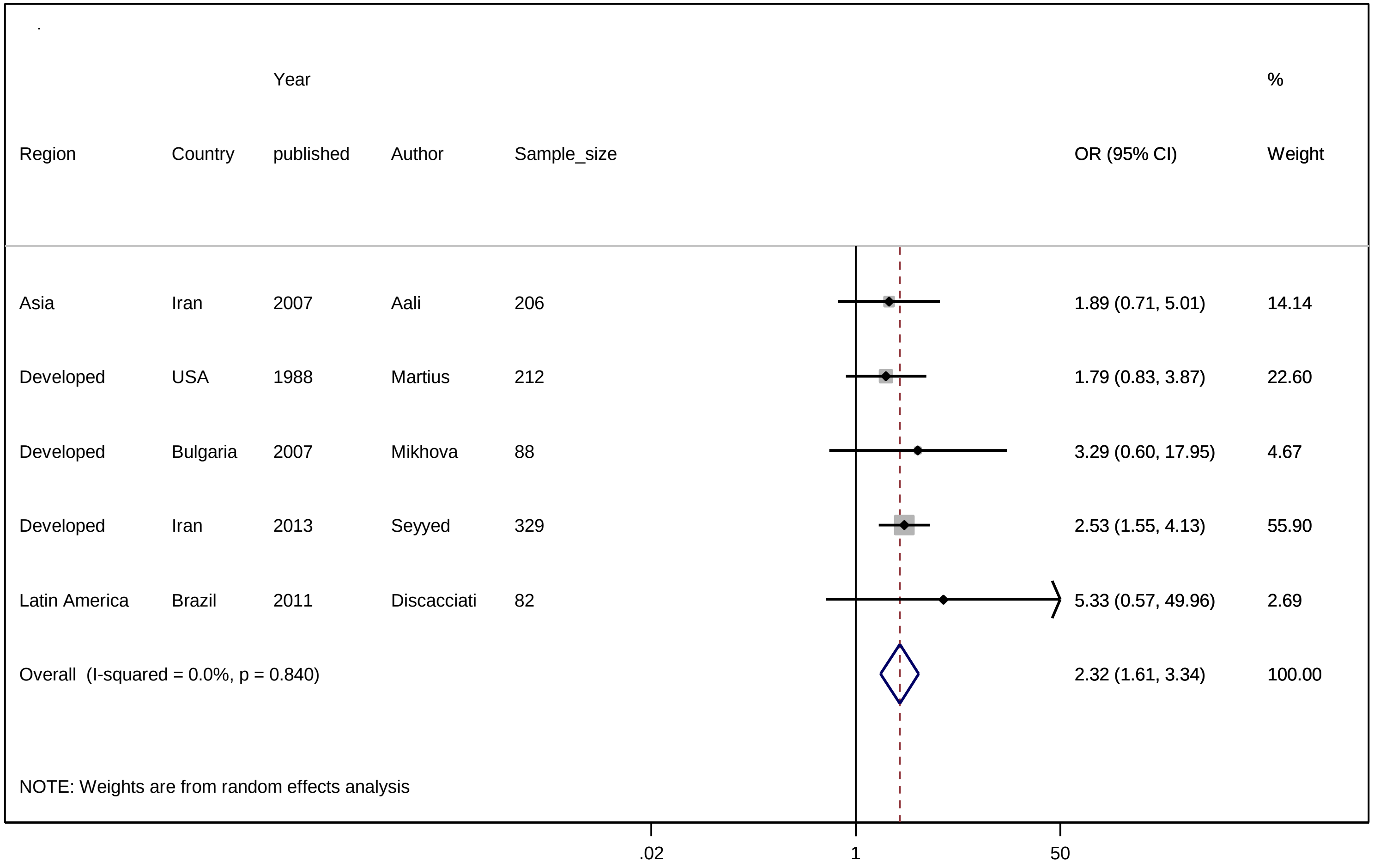

Supplementary Figure S9: Meta-analysis of cohort and cross sectional studies for studies measuring GA by LMP, ultrasound and/or fundal height

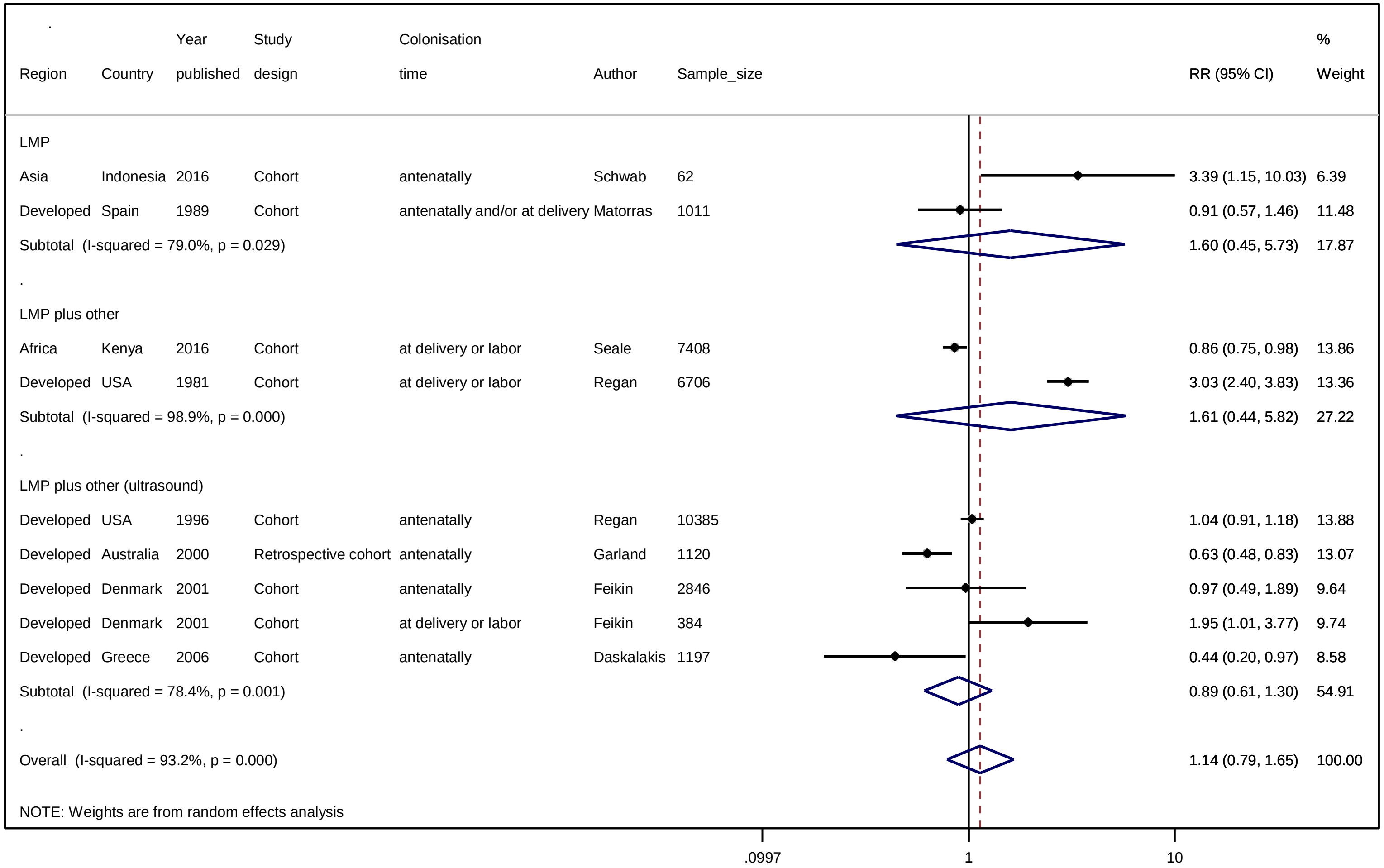

Supplementary Figure S10: Meta-analysis of case-control studies for studies for studies measuring GA by LMP, ultrasound and/or fundal height

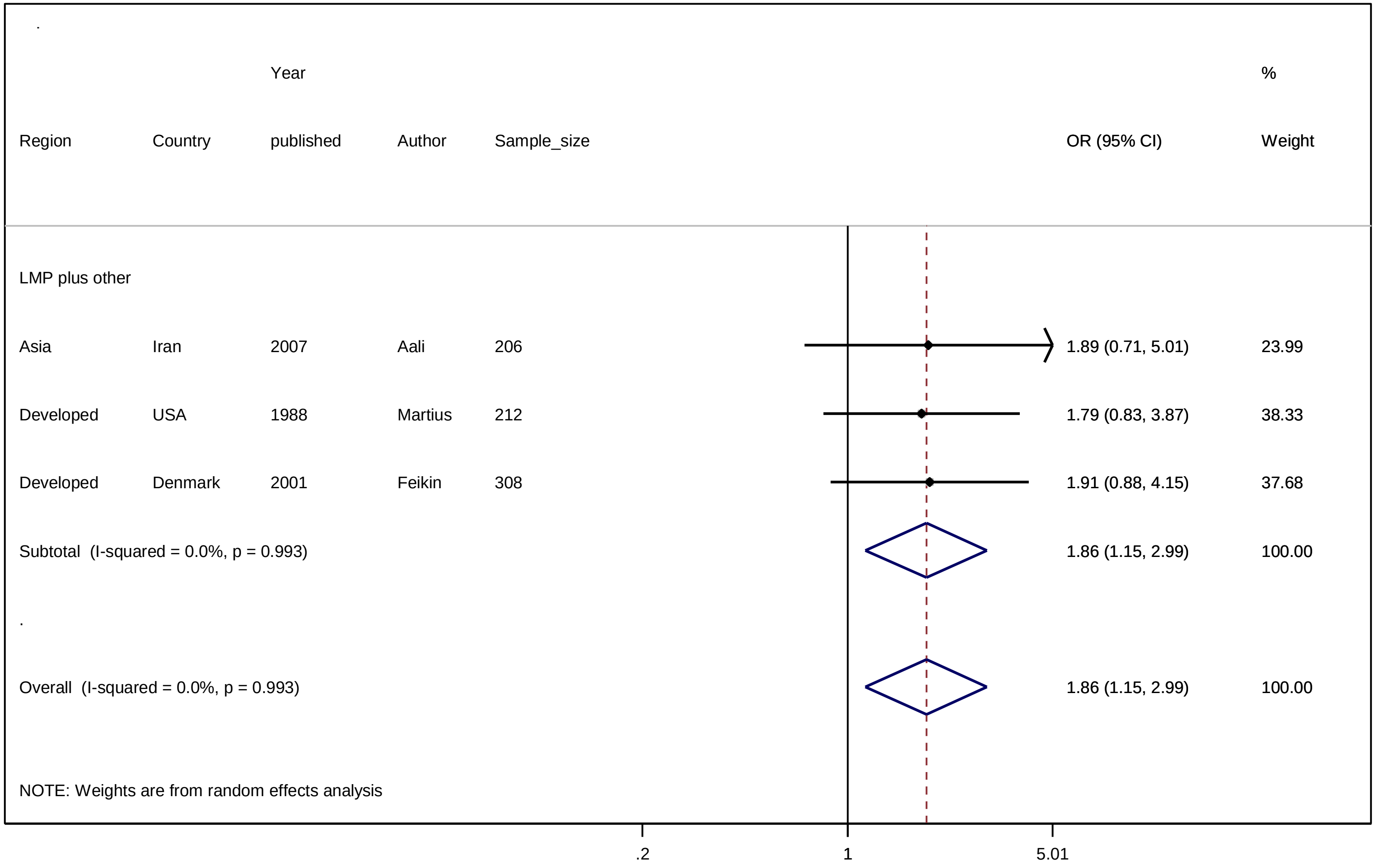

Supplementary Figure S11: Meta-analysis without cohort and cross-sectional studies using different thresholds for the definition of preterm (or not specifying the definition) by time of sampling

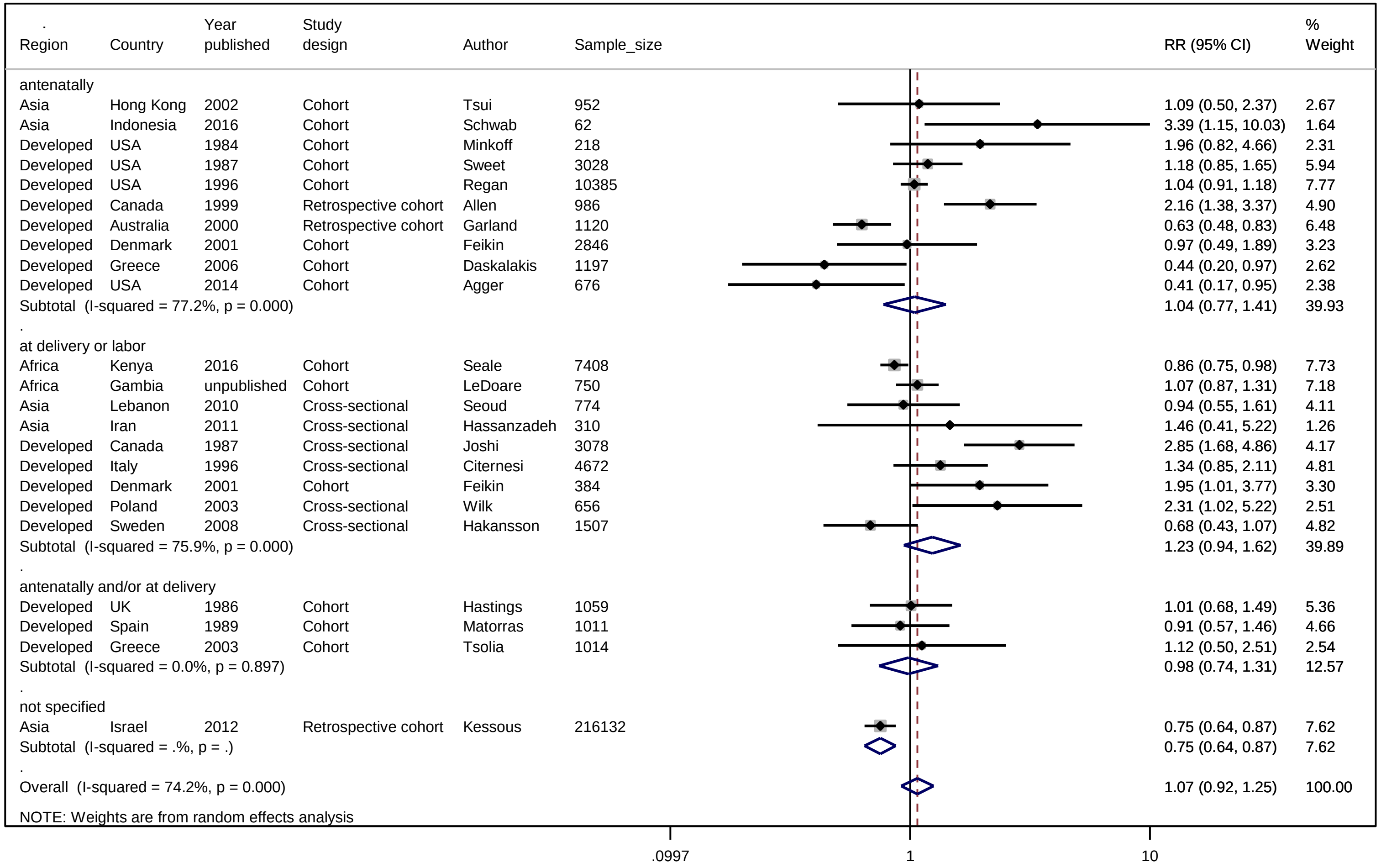

Supplementary Figure 12: Meta-analysis without case-control studies using different thresholds for the definition of preterm (or not specifying the definition)

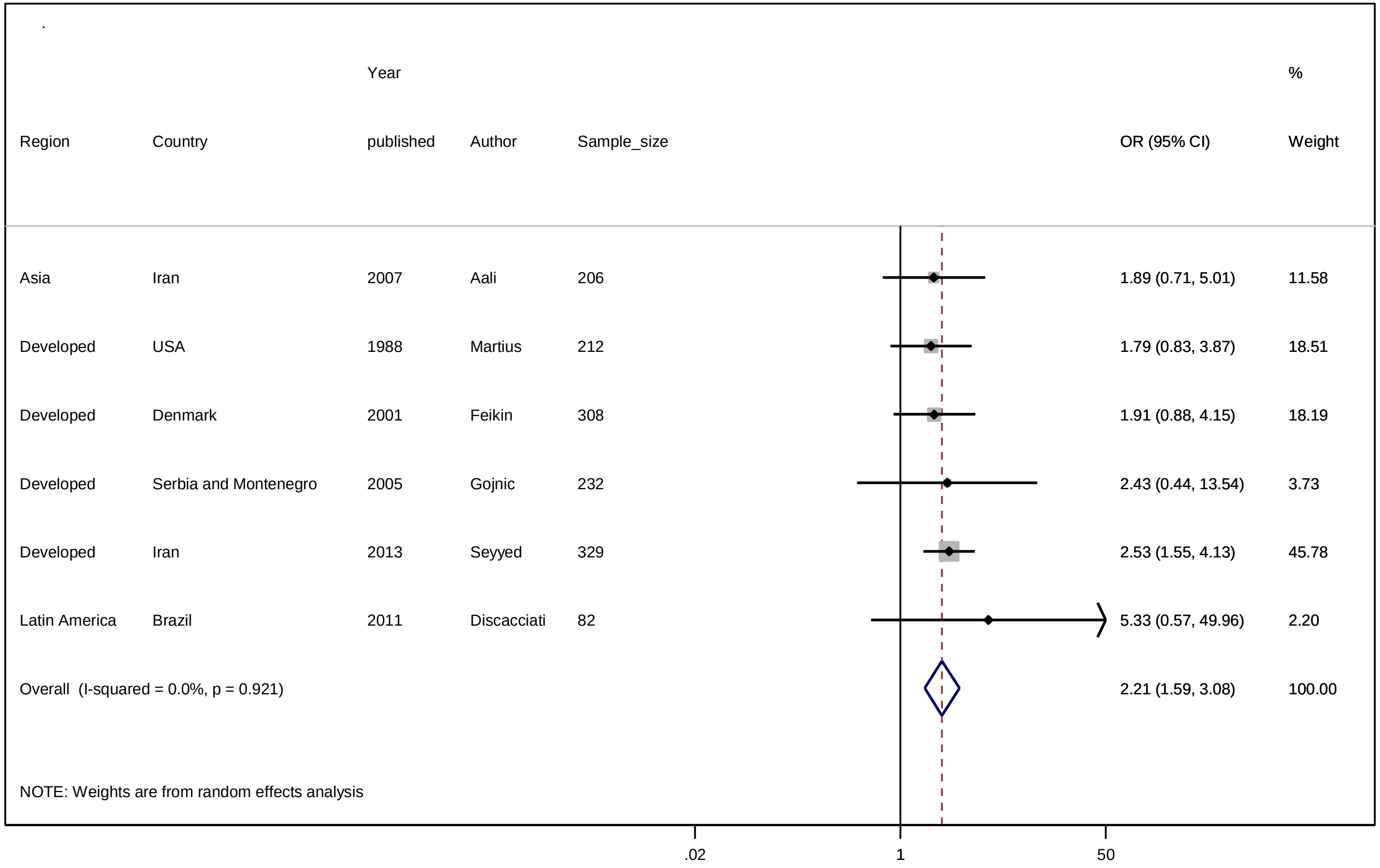

Supplement: supplement-material [file cix661_suppl_supplement-material.pdf]
